# The Effect of a Health Game Prompt on Self-efficacy: Online Between-Subjects Experimental Survey

**DOI:** 10.2196/20209

**Published:** 2021-03-03

**Authors:** Priscilla Haring

**Keywords:** self-efficacy, games for health, serious games, arousal congruent, cognitive reappraisal, prompt, flourishing, eHealth, diabetes

## Abstract

**Background:**

Games for health are increasingly used as (part of) health interventions and more effect research into games for health is being done. This online experiment questions expectancies of games for health by investigating whether a game for health prompt might be considered arousal congruent cognitive reappraisal and as such positively effects self-efficacy before gameplay.

**Objective:**

The aim of this study experiment is to test whether a game for health prompt effects self-efficacy and other well-being measurements, as a first step into investigating if a game prompt is a form of arousal congruent cognitive reappraisal.

**Methods:**

This study used an online, 2D, between-subjects experimental survey design with self-efficacy as the main dependent variable. Stimulus is an assignment for health-related problem solving concerning living with diabetes type II, introduced as a game (n=125) versus the same assignment introduced as a task (n=107). Measurements after prompting the game/task assignment include self-efficacy, positive and negative affect, expected difficulty, flourishing, and self-esteem.

**Results:**

The results indicate a small negative effect from prompting the game assignment on self-efficacy, compared with prompting a task assignment. This effect is mediated by the expected difficulty of the health game/task. No differences between the game and task groups were found in affect, flourishing, or self-esteem.

**Conclusions:**

This experiment provides no support for the notion that a game for health prompt might be seen as arousal congruent cognitive reappraisal.

## Introduction

### Origin

In “Get excited: Reappraising pre-performance anxiety as excitement” by Brooks [1], the concept of arousal congruent cognitive reappraisal is examined by offering someone in an anxious state a prompt that aims to change their negative affect but leaves their arousal high (Table 1). She does this through prompting participants with “I am excited” and subsequently participants performed better on the task that made them anxious, and experienced a higher sense of self-efficacy. The idea is that it is more effective to shift from negative to positive affect when you can maintain your level of arousal. This follows a new line of thinking in stress research, which suggests that stress—a state of high arousal—is not necessarily harmful; effects depends on how we interpret stress. If we think and feel positive about our high arousal, stress can be helpful to our health [2].

While reading the work of Brooks, it seemed to me that prompting a game for health is also a call to be excited amidst anxious content and I wondered if a game for health might be considered arousal congruent cognitive reappraisal? If so, this perspective could help to explain some of the attraction to games for health and their effects on self-efficacy.

**Table 1:** Excitement is a state of high arousal and positive affect. Anxiety is a state of high arousal and negative affect.

Affect	High arousal	Low arousal
Positive	Excited	Calm
Negative	Anxious	Bored

Research question: Is prompting a game for health a form of arousal congruent cognitive reappraisal?

### Games for Health

Health science has been embracing gaming as a meaningful way to communicate, educate, and as a mechanism to deliver treatment [3,4]. There has been a growing interest in both serious games for health and gamified health interventions [5], especially those concerning the treatment, rehabilitation, and management of chronic disease patients [6], as these games for health have shown potential for positive impact on health-behavior change [7].

Some research on the effectiveness of serious gaming for health promotion revealed an overall increase in healthy lifestyle adoption across several health domains [4] and that gameplay may induce a positive emotional experience and help facilitate satisfaction and self-esteem [6]. Effect sizes found on behavior after playing a serious game were small and comparable to the effect sizes of other computer-delivered interventions. Such effects of serious games for health were highest on knowledge outcomes, while smaller than expected on self-efficacy outcomes. Overall, the effectiveness of a health game was found to improve when game development had a theoretical foundation in behavioral prediction and game theories [8].

In order to create a full picture of the effectiveness of games for health, broader intervention characteristics should perhaps be evaluated, such as user experience and perceived relevance [9]. Some of the research into the effectiveness of games for health investigates the process during gameplay [10,11] while other research focuses on process after gameplay [8,12]. I would argue that the perceived appeal of any game for health belongs in this list of “broader intervention characteristics” and that measurements should also be made BEFORE gameplay. In this experiment a game (or a task) is announced but not given, and all the measurements concern expectations of gameplay (or task performance) that is yet to come.

### Cognitive Reappraisal

Cognitive reappraisal is a change in cognition which allows for the interpretation of an emotion-eliciting situation in such a way as to alter the emotional impact it has [13]. It is using what you think to change what you feel. Cognitive reappraisal was found to increase cortisol reactivity in both a public speaking task and a cold pressor pain task, which suggests that cognitive reappraisal might even support greater physiological reactivity to acute stress and that it may increase active coping strategies [14]. One experiment showed the simplicity of cognitive reappraisal by announcing an anxiety-inducing math test as a “challenge.” This decreased the experienced threat of the test and improved math performance among both high-school and university students [15]. Cognitive reappraisal might also be an effective strategy for mitigating the effects of experiencing anxiety from health-related messages. Health-risk information can be threatening in its nature and induce defensive responses [16], and health appeals can be interpreted as threatening as they confront us with disease and our own mortality [17]. The resulting state anxiety can drain working memory capacity, decrease self-confidence, harm task performance [18], and has been linked to a lowering in self-efficacy [19]. These processes are especially pertinent in a health environment, as on the one hand health information can elicit negative emotions, while on the other hand self-efficacy is known to play a role in achieving effective health behaviors [20].

### Self-efficacy

Self-efficacy is a persons’ belief in their capability to perform any task [19]. We tend to interpret our physical responses and our affective state as related to our capabilities, while this need not be the case. Persons with a high sense of self-efficacy can interpret a state of arousal as a motor to action, whereas persons with a low sense of self-efficacy can interpret the same state as an obstacle to action, or even an indication to cease all efforts [1]. Research has shown that self-efficacy is an important construct in many health behaviors, and it is widely seen as an important part of creating short- and long-term changes in health-related behavior [20]. A higher sense of self-efficacy has been linked to lower physiological stress responses [21] and better adherence to medical treatment [22,23].

Playing games for health has shown to invoke positive feelings [6] and improve self-efficacy [8]. Arousal is seen as a vital part of the attraction of gaming [24] and a good game will keep you aroused and engaged throughout. These elements (positive affect and high arousal) suggest that playing games for health (anxiety-inducing context) might be a form of arousal congruent cognitive reappraisal, and I suggest that this reappraisal starts before gameplay begins at the point of announcing the game. The more difficult the announced context is expected to be, the stronger the effect of reappraisal by the game prompt should be on self-efficacy.

H1 Prompting a game for health will increase self-efficacy, when compared to prompting a task for health.

H2 Prompting a game for health will increase positive affect and decrease negative affect which will correlate with higher self-efficacy, when compared to prompting a task for health.

H3 Difficulty judgment will positively mediate the strength of the correlation between prompting a game for health and self-efficacy.

### Flourishing and Self-esteem

Besides affect, flourishing and self-esteem are also incorporated as a measure of well-being that are connected to self-efficacy. The concept of flourishing [25] is based on investigating optimal human functioning; it incorporates several constructs from the field of positive psychology [26]. In contrast to the commonly used hedonic approach of subjective well-being, flourishing is based on the *eudaimonic* approach of psychological well-being and involves some of the same constructs as self-efficacy from the mastery perspective [27]. Participants reporting a higher sense of flourishing are expected to have a higher score on self-efficacy. Higher self-esteem is also linked to higher levels of mastery and self-efficacy [28]. “Self-esteem can be defined as an evaluation of one’s self-concept, which is heavily dependent on reflected appraisals, social comparisons, and self-attribution” [29]. Although global self-esteem has shown to be related to many self-evaluations, it is not always equal to any domain-specific self-evaluation [30] and the effect of self-esteem on self-efficacy in this case remains to be seen. Games have been shown to facilitate greater self-esteem in a health-related context [6] and in this research I am interested in finding out if this facilitation starts at the expectancy of playing a game.

H4 Prompting a game for health will increase flourishing and self-esteem which will correlate with higher self-efficacy, when compared to prompting a task for health.

### Diabetes Context

The health-related context for this experiment is that of “living with diabetes type II” which is selected as it is a prevalent lifestyle disease that is greatly impacted by health behavior, and health professionals have been especially interested in video games as a way to deliver diabetes self-management support [31-33]. Diabetes research has identified self-efficacy regarding self-care as a pivotal psychosocial variable, showing correlations of self-efficacy scores with self-care behaviors in diet, exercise, and blood glucose testing [20]. Some studies involving the effect of games for health have specifically measured the effect of a game on self-efficacy as well as behavior. One famous study investigated the effects of *Packy and Marlon* [34], which is a video game for children with diabetes where the main characters must manage their insulin levels and food intake while protecting other game characters from a rat infestation at a summer camp. After playing *Packy and Marlon* for 6 months, players not only displayed more knowledge of diabetes and its treatment, but they also displayed greater self-efficacy in management of the disease and showed improvement in diabetic-related health behavior [35]. A more recent paper described 14 different diabetes self-management games that often simulate problem solving of trying to balance food, insulin, and blood glucose. Although most games do not provide clinical validation, they are shown to improve behaviors that support diabetes self-management and will lead to better health outcomes [31].

### Model of Expected Relation Between Variables

**Figure 1:**
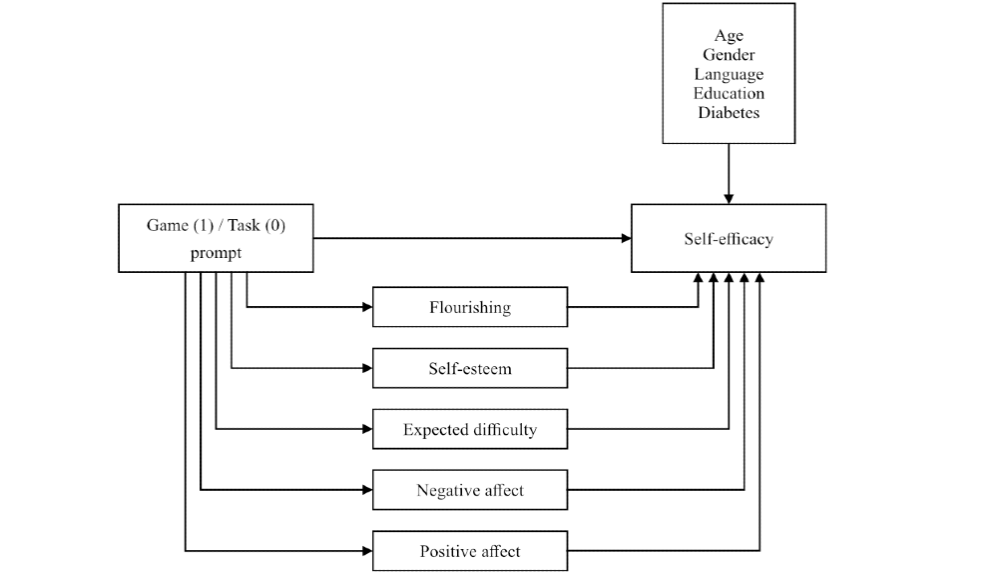
Hypothesized relationships.

As you can see in Figure 1, I expect that participants prompted by a game for health will have higher scores on self-efficacy (H1). I also expect that positive and negative affect (H2) expected difficulty (H3), and both flourishing and self-esteem (H4) will all partially mediate the effect of a game prompt on self-efficacy. Demographic information such as age, gender, education, English as first language, and familiarity with diabetes is expected to correlate with self-efficacy, but not with receiving a game or task prompt.

## Methods

### Study Design

This is an online, 2D, between-subjects experimental design with self-efficacy as the main dependent variable.

### Stimulus

The stimulus material contains references that are both health related and math related, in an attempt to increase a sense of difficulty. The opening page of the survey displayed the blue logo of the Diabetes Foundation and 2 blue snakes around a blue staff, a visual that is generally associated with the medical profession. On this page participants were confronted with a text asking them to solve issues related to living with diabetes type II. The text tells them that they will have to solve these unknown problems in either a *game* or in a *task* environment:

Thank you for participating in this research, it will take approximately 5-10 minutes. All your answers will be stored and analysed anonymously.

HEALTH GAME [TASK] On the next pages you will be asked to play a game [task] in which you have to solve several health related problems concerning living with Diabetes type II. This game [task] includes dealing with glycaemic control, caloric intake, measurement intervals and other issues. The game [task] requires no specific prior knowledge and you do not need to have Diabetes to participate.

After reading the text, the survey begins on the next page. Throughout the survey the words “game” or “task” are repeated 12 times. A manipulation check is included after the measurements, asking the participants if they are about to play a game or perform a task (or they don’t know). At the end of the survey a short debriefing explains the purpose of the experiment.

### Participants

To establish the necessary number of participants, a power calculation was performed with G*Power [36]. A 2-tailed, a priori power calculation for *t* test mean difference between 2 independent groups was done, with the expected effect size of the dependent variable based on “[…] participants in the “get excited” condition reported higher self-efficacy by comparison (mean 5.66 [SD 1.01], *t*=–2.35, *P*=.021; *d*=.415)” [1]. This power calculation indicated that 186 or more participants would be sufficient to detect the expected effect of *d*=0.415 with a power of (1–β)=0.8 and α=.05, when N=186 is equally distributed over the 2 independent groups. Random and even distribution of participants into either the game or task group was managed by Qualtrics, the software used for the survey design and online data collection [37].

**Figure 2:**
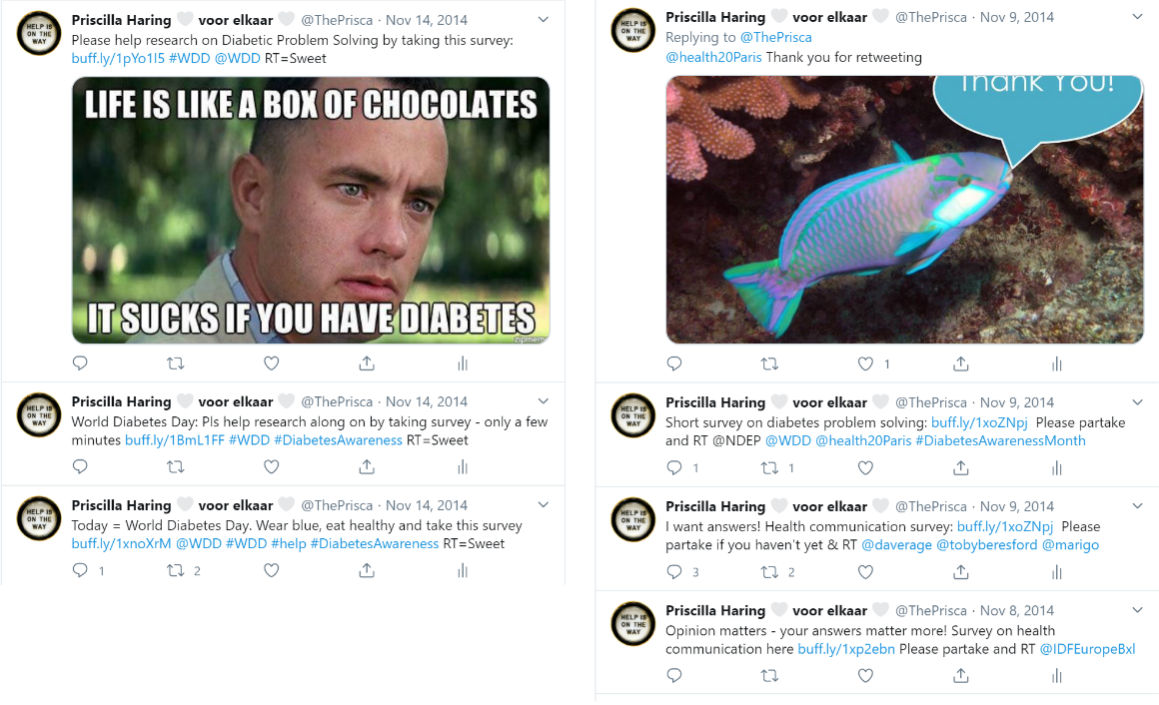
Recruiting participants on Twitter.

The experiment was hosted online for 2 weeks during the Diabetes Awareness Month. A post on my social media accounts invited people to participate in “research on health-related choices” and encouraged sharing a link to the research with others. Participants were also recruited by specifically targeting Twitter accounts that were game and diabetes related (Figure 2). There were no entry criteria and no consequences to participating.

In a second round participants were hired on the micro-task market: Amazon’s Mechanical Turk. Such Mechanical Turks are best used in cases where there is a small task with a need for many users, when there is a verifiable answer, and there are no objections to a diverse and unknown group of participants [38]. To encourage accurate answers, I included an announcement before the survey started that a check (the manipulation check) was included in the survey, and that there would be no pay-out if this check was missed or wrong. These MTurks were paid the equivalent of 8 minutes’ work under Dutch adult minimum wage. Eventually, 78 participants in the data set (33.6%) originated from online snowballing and 154 (66.4%) were recruited as MTurks (N=232).

Participants that passed the manipulation check and had no missing values were included in the analysis. In total, 232 participants were included in analysis (115 men and 117 women; average age 37.5 years, 125 game group and 107 task group). No significant differences were found between the game and task group on the variables gender, age, education level, or experience of diabetes.

### Measurements

The survey consisted of 4 screens. The first screen contained the introduction and the stimulus, the second contained the self-efficacy and expected difficulty measurement. The third screen contained demographic, flourishing, Positive and Negative Affect Schedule (PANAS), self-esteem, experience of diabetes measurement, and the manipulation check. The fourth and last screen contained the debriefing and a comment/question box, followed by my thanks, my name, and academic title.

#### Self-efficacy

In this experiment self-efficacy is measured by adapting 2 established measures: the 13-item reduced form Coping Self-Efficacy Scale (CSES) [39] and the State Self-Efficacy Subscale (SSESS) [40]. The base question for the CSES was adapted to the context of the experiment:

“Before we start - We want to ask you to give a confidence rating on the game you are about to do. How confident or certain are you that you can do the following things in the game on Living with Diabetes type II”. This phrasing is in line with the ‘Guide for constructing self-efficacy scales’ [41].

This experiment uses 2 CSES subscales: Problem-Focused Coping (PFC; 6 items, α=.90) and Stop Unpleasant Emotions and Thoughts (SUET; 4 items; α=.90).

Items of the CSE subscale PFC include the following:

- Break an upsetting problem down into smaller parts
- Sort out what can be changed, and what cannot be changed
- Make a plan of action and follow it when confronted with a problem
- Leave options open when things get stressful
- Think about one part of the problem at a time
- Find solutions to your most difficult problems

Items of the CSE subscale SUET include the following:

- Make unpleasant thoughts go away
- Take your mind off unpleasant thoughts
- Stop yourself from being upset by unpleasant thoughts
- Keep from feeling sad

For this experiment, the wording of the items from the SSESS was adapted from the evaluative form into an expectant form. Furthermore, 2 items based purely on self-efficacy of content knowledge were deleted, leaving 4 items (α=.94).

Items of the adapted SSESS include the following:

- In the GAME/TASK, I expect to do well
- I have no doubts about my capability to do well on this GAME/TASK
- I am sure I can do an excellent job in this GAME/TASK
- Even when the GAME/TASK questions are difficult, I know I can succeed

**Figure 3:**
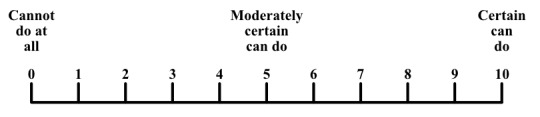
Self-efficacy answer format.

All 14 self-efficacy items are combined in 1 matrix (α=.95). Performing a principal component factor analysis on the full matrix of 14 items revealed 1 underlying component. All answers were given on a 0-10 range with 3 semantic anchors (Figure 3), the same answer format that is used in the CSES.

#### Positive and Negative Affect

In order to measure emotional valence, the PANAS is used [42]. When Kobau and colleagues [43] researched the most used measurements of well-being as related to health, they found an interitem consistency of α=.91 for the Positive Affect Subscale and α=.86 for the Negative Affect Subscale.

#### Flourishing

Another measure of well-being is taken with the 8-item Flourishing Scale [26] with answers given on a 5-point Likert scale from 1 (strongly disagree) to 5 (strongly agree). This relatively new scale was also used as part of the New Zealand’s Sovereign Well-Being Index (N=100,009) [44]. Subsequent analyses of the underlying structures and psychometric properties of the scales were performed as well as reliability and validity checks and benchmarking to other well-being scales used in the survey. The study concluded that the flourishing scale “is a valid and reliable brief summary measure of psychological functioning, suited for use with a wide range of age groups and applications ” [44]. In the data from this experiment, the flourishing scale (α=.90, mean 4.07 [SD 0.65]) correlates with other measurements of well-being (PANAS and self-esteem) as expected.

#### Expected Difficulty

The expected difficulty of the game or task is measured in 1 item by asking the participants how difficult they expect the task/game is going to be and assessing this on a 7-point scale with 2 semantic anchors (1=very easy, 7=very difficult). This measure is an adaptation of the After-Scenario Questionnaire [45].

#### Self-esteem

Self-esteem is measured by a Single-Item Self-Esteem (SISE) Scale. The SISE was banked against the Rosenberg Self-Esteem Scale [46] along with other measures of domain-specific self-evaluation, personality, well-being, and behavioral measures and was found to give a reliable measure of self-esteem for adults [30].

#### Diabetes Experience

Participants were asked how familiar they were with diabetes and its challenges, choosing between 4 possible answers:

- I have Diabetes
- Someone close to me has Diabetes
- I have no personal experience of Diabetes but I am aware of what Diabetes is and what the challenges are
- I have no or very limited knowledge on this subject

#### Demographics

Participants were also asked about their gender, level of education, year of birth, and if their native language was English or otherwise.

## Results

### Overview

The research question investigates the idea that prompting a game to solve health-related problems might be a form of arousal congruent cognitive reappraisal, similar to the “get excited” prompt [1] and as such would result in increased self-efficacy. In order to start exploring this question, a number of hypotheses were formed and tested. If the announcement of a game for health is arousal congruent cognitive reappraisal, participants that are prompted by such a game for health are expected to have higher scores on self-efficacy (H1) while positive and negative affect (H2), expected difficulty (H3), and both flourishing and self-esteem (H4) will all partially mediate the effect of a game prompt on self-efficacy, compared with participants that are prompted by a task for health.

### Correlations of Measurements

Looking at the participants in either the game or task group in Table 2, there is a small, negative correlation between self-efficacy and belonging to the game or task group (*r*=–.17, *P*<.01) (H1) as well as a small, negative correlation between belonging to the game or task group and difficulty judgment (*r*=–.15, *P*<.05) (H4). However, no other significant correlations are found. Self-efficacy scores also correlate significantly with positive affect (*r*=.49, *P*<.01) and negative affect (*r*=–.35, *P*<.01) (H2), difficulty judgment (*r*=.40, *P*<.01) (H3), flourishing (*r*=.54, *P*<.01), and self-esteem (*r*=.41, *P*<.01) (H4).

Further negative correlations with self-efficacy scores are shown with diabetes knowledge (*r*=–.22, *P*=.001) and being an English native speaker (*r*=–.13, *P*=.04), while a positive correlation is being shown with being recruited as an MTurk (*r*=.29, *P*=.001) versus via snowballing.

**Table 2:** Correlation table.

Correlation	Positive affect	Negative affect	Self-efficacy	Difficulty judgment	Flourishing	Self-esteem	Diabetes experience	Education	Gender	Age	Language	MTurk
Game (1)/Task(0)	–.100	.007	–.170^b,c^	–.150^a,b^	–.125	–.044	.087	–.035	–.070	.007	.020	–.091
Positive affect	—	–.143^a,b^	.494^b,c^	.365^b,c^	.594^b,c^	.522^b,c^	–.236^b,c^	.155^a,b^	–.068	–.001	–.046	.312^b,c^
Negative affect		—	–.346^b,c^	.074	–.385^b,c^	–.155^a,b^	.003	.015	–.052	–.221^b,c^	.116	.004
Self-efficacy			—	.404^b,c^	.543^b,c^	.411^b,c^	–.224^b,c^	.035	.007	.000	–.133^a,b^	.289^b,c^
Difficulty judgment				—	.303^b,c^	.390^b,c^	.200^b,c^	.083	–.108	–.195^b,c^	.003	.340^b,c^
Flourishing					—	.569^b,c^	–.183^b,c^	.138^a,b^	.088	–.020	–.080	.142^a,b^
Self-esteem						—	–.189^b,c^	.191^b,c^	–.099	–.045	–.130^a,b^	.296^a,b^
Diabetes knowledge							—	.102	.055	–.224^b,c^	.325^b,c^	–.325^a,b^
Education								—	–.014	–.148^a,b^	.266^b,c^	–.113
Gender									—	.043	.038	–.122
Age										—	–.114	–.095
Language											—	–.391^b,c^

^a^*P*<.05 (2-tailed), N=232.

^b^Significant value.

^c^*P*<.01 (2-tailed).

### Direct Effect of Belonging to the Game or Task Group on Self-efficacy

To investigate the effect of prompting a game or task for health on self-efficacy (H1), a comparison was made of the average compound self-efficacy score (14 items) of the game versus the task group via independent samples *t* test. This analysis revealed a difference in self-efficacy scores (*t*_230_=2.62, *P≤*.01) between the game group (mean 8.08 [SD 1.75]) and the task group (mean 8.66 [SD 1.61]) in the direction opposite to expectations, showing a small but significant (*P≤*.01) higher sense of self-efficacy in the task group. Performing the same *t* test on standardized self-efficacy scores reveals a small to medium effect size of *d*=3.41.

To investigate the relation between belonging to the game or task group and self-efficacy further, the average scores on these (sub)scales between the game or task group were tested simultaneously by a one-way between-subject ANOVA. For the SSESS significant differences between the game group (mean 8.12 [SD 2.21]) and the task group (mean 8.97 [SD 1.72]) were found, with the Levene test showing that the variances of these scores were not equal (*F*_1,230_=10.91, *P*=.001). Participants also scored differently on the subscale CSE-PFC in the game group (mean 9.77 [SD 2.11]) versus the task group (mean 10.45 [SD 1.92]; *F*_1,230_=6.58, *P*<.05). However, on the subscale CSE-SUET between the game group (mean 7.94 [SD 2.14]) and the task group (mean 8.27 [SD 2.01]) no significant differences could be found (*F*_1,230_=1.50, *P*=.22).

### Direct Effect of Belonging to a Game or Task Group on Affect

To investigate if the game group will have a lower negative affect and a higher positive affect when compared with the task group (H2), an independent sample *t* test was performed to compare average affect scores between groups. The scores on positive affect in the game group (mean 3.54 [SD 0.81]) and the task group (mean 3.69 [SD 0.70]) showed no significant difference (*t*_230_=1.53, *P*>.1). The scores on negative affect in the game (mean 1.83 [SD 0.78]) and task groups (mean 1.82 [SD 0.82]) also were not significantly different (*t*_230_=–0.10, *P*=.92).

### Effect of Difficulty Judgment on the Relation between the Game or Task Group and Self-efficacy

Investigating the subset of participants that answered Somewhat difficult, Difficult, or Very Difficult (n=79), a difference on the average self-efficacy score (H3) between the game (mean 7.00 [SD 2.38]) and task (mean 8.52 [SD 2.16]) prompted group was found, but this was not significant (*t*_77_=1.72, *P*>.05).

### Direct Effect of Belonging to the Game or Task Group on Flourishing and Self-esteem

To investigate if belonging to the game group has a positive effect on flourishing (H4), an independent sample *t* test was performed to compare average flourishing scores between the game and task groups. The scores on flourishing in the game group (mean 4.00 [SD 0.58]) and the task group (mean 4.16 [SD 0.69]) showed no significant difference (*t*_230_=1.91, *P*=.58). Comparing the average self-esteem scores (H4) between the game group (mean 3.79 [SD 1.02]) and the task group (mean 3.87 [SD 0.95]) also showed no significant differences (*t*_230_=.67, *P*=.51).

### Demographics and Diabetes Experience

No significant differences were found between participants in the game or task group on age (*t*_230_=–0.11, *P*=.92), gender (*t*_230_=1.06, *P*=.29), language (*t*_230_=–0.30, *P*=.77), education level (*t*_230_=0.53, *P*=.60), or experience of diabetes (*t*_230_=–1.32, *P*=.19).

### Mediation Model Test

**Figure 4:**
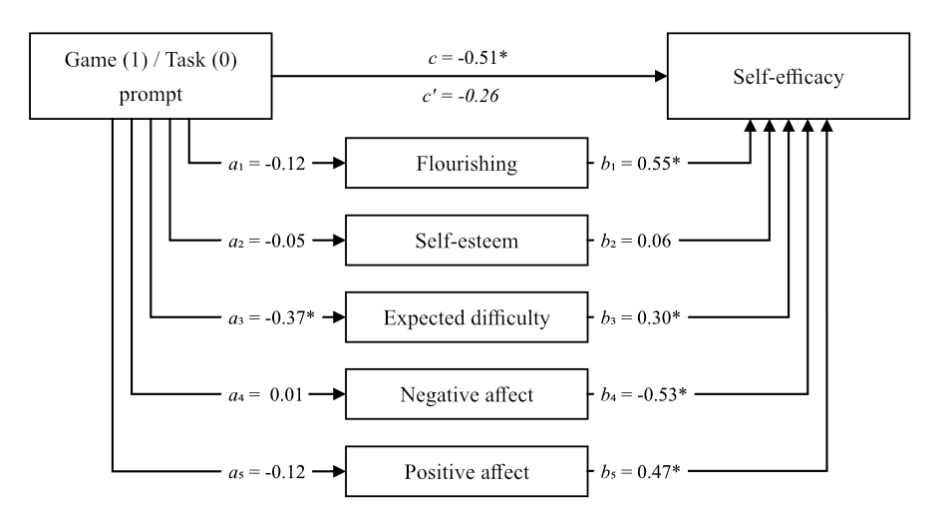
Mediation model. Multiple mediator model with flourishing, self-esteem, expected difficulty, and both negative and positive affect as mediators on the effect of a game or task prompt on self-efficacy. Age, gender, language, education, and diabetes experience are included as covariates (paths not shown). *P<.05.

In order to test the conceptual model as a whole, a mediation analysis was conducted using the PROCESS macro (model 4: 10,000 bootstrap samples). This model is used for both simple mediation models and parallel multiple mediator models [47]. The effect of belonging to the task or game prompt group on self-efficacy was tested, while including positive and negative affect, difficulty judgment, self-esteem, and flourishing as possible mediators and adding diabetes knowledge, first language, age, education, and gender as covariates. The results are shown in Figure 4.

This analysis shows that the direct effect path between a game and a task prompt on self-efficacy is significant (*c*=–0.51, 95% CI –0.94 to –0.07), but this effect loses strength and its significance when the mediators are taken into account (*c*′=*–*0.26, 95% CI –0.61 to 0.08). In the model expected difficulty was shown to be a mediator, as both the path from game or task prompt to expected difficulty (*a*_3_=*–*0.37, 95% CI –0.71 to –0.02) and the path from expected difficulty to self-efficacy (*b*_3_=0.30, 95% CI 0.16 to 0.45) were significant. For flourishing (*b*_1_=0.55, 95% CI 0.16-0.94) as well as negative affect (*b*_4_=0.53, 95% CI –0.77 to –0.29) and positive affect (*b*_5_=0.47, 95% CI 0.18-0.77) only the second paths in the model show a significant effect, indicating that these might be moderating but not mediating the relationship between the game or task prompting and self-efficacy. Self-esteem on both paths (*a*_2_=0.05, 95% CI –0.29 to 0.20 and *b*_2_=0.06, 95% CI –0.17 to 0.29) shows no effects of significance in this model.

Further testing of flourishing and positive and negative affect as moderators between game or task prompting and self-efficacy (including the same covariates as in the mediation model) did not result in any significant paths, and discounted these variables as moderators.

### MTurks or Snowballing

Average scores on several variables between the 2 differently recruited groups were compared and tested via independent *t* tests. MTurk participants showed a higher score on the self-efficacy measure (*t*_230_=4.582, *P*≤.001, *d*=0.61) when compared with the participants that were recruited via snowballing. MTurk participants expected the task/game to be less difficult (*t*_230_=5.486, *P*≤.001), in comparison to the snowballing group. The group of MTurks also scored significantly higher on flourishing (*t*_230_=2.172, *P*≤.05), the positive part of the PANAS (*t*_230_=4.974, *P*≤.001), self-esteem (*t*_230_=4.696, *P*≤.001), and indicated more experience of diabetes (*t*_230_=–5.026, *P*≤.001).

### Data

The anonymized data set “The Effect of a Game Prompt on Self-Efficacy Concerning Problem-Solving Challenges of Living with Diabetes type II” can be found online at the Open Science Framework [48].

## Discussion

### Principal Findings

There are several significant correlations between the variables measured in this online survey. Being confronted with the game for health stimulus correlates with a little less self-efficacy and with the expected content being judged a little more difficult, compared with being confronted with the task stimulus. The scores of the flourishing scale correlate with the PANAS, self-esteem, and self-efficacy as expected, confirming these measurements of well-being among themselves within this data set.

When the relationship between self-efficacy after a game prompt or a task prompt was tested, scores in each group are significantly different. The game group scores an average of 8.08 on self-efficacy, while the task group scores on average 8.66. This scoring indicates answers between 0=cannot do at all and 11=certain can do. Even though a difference in scoring between groups is found, the difference in scores is small and the average scoring in both groups represents a high amount of self-efficacy.

A significant difference between game and task group participants holds on the State Self-Efficacy Scale and PFC Subscale, but disappears in the scores on the subscale for stopping unwanted emotions or thoughts. That no effect could be found on this subscale might be due to the limited timespan of the survey (5-10 minutes) which is likely not long enough to raise the issue of consciously controlling ones’ emotional and cognitive state.

The expectation that a game for health prompt would be followed by more positive affect and less negative affect was not found. No significant difference in affect was found at all between the game and task groups. Neither could any significant difference in the scores on self-esteem or flourishing be found between the task and game groups.

When looking at the subset of participants that judged the expected content to be difficult, no significant effect of either the game or task stimulus could be found on self-efficacy. However, because many participants did not judge the expected content as difficult, this analysis relies on a smaller number (n=79) which might explain the lack of a robust finding. The results might be indicating a trend that a drop in self-efficacy following a game prompt will get bigger if the content is expected to be more difficult.

Through running a mediation analysis it becomes clear that the difficulty judgment fully mediates the connection between participating in the game prompt or task prompt group and the score of self-efficacy. This mediation indicates that a game for health prompt creates the expectancy of slightly more difficult content compared with the task prompt, which influences the relationship between the type of prompt and self-efficacy in the direction of a game prompt being followed by a little less self-efficacy.

No differences were found between the game and task group in flourishing and self-esteem average scores. Although negative and positive affect as well as flourishing show a significant relation to self-efficacy scores in the mediation model, neither mediation nor moderation can be established and not much can be said on the connection between these variables from these data.

The use of MTurks for online surveys and experiments is getting more widespread [49]. However, this experiment shows that how participants are recruited can have its own effect on outcomes. On all the self-evaluative measures in this experiment, MTurks scored different from the snowball participants. How participants were recruited showed a greater effect on self-efficacy (*d*=.61), the main dependent variable in this study, than the effect of the manipulation (*d*=.34).

### Limitations

This experiment has no heart rate measure as an indication of arousal, which is a practical limitation of doing online research. Subjective measures of arousal do exist (such as a self-report scale that might be used online), but research indicates that such measures did not match physiological data collected via electromyography and skin conductance [50] and as such do not provide valid measurements.

Although mediating effects are found from difficulty judgments, participants on average expected this assignment to be “neutral,” meaning neither difficult nor easy. Future research might investigate game or task prompting where the judgment of the expected content is on the “very difficult” side of the scale.

No measurement of game literacy is included in the study; this information might provide interesting correlations with expectations of playing games for health. Future research might look at the level of experience with 3 game categories: entertainment games, serious gaming, and games for health. A further investigation of lay-beliefs and expectancies of these 3 categories before any gameplay seems warranted.

### Conclusions

The aim of this study was to try and establish a first foothold into investigating whether prompting a game for health might be considered arousal congruent cognitive reappraisal. As far as this one small study can indicate anything, it appears to indicate that this is not the case. Games or gamification in health care context have been shown to increase self-efficacy [6]. However, this positive process does not seem to start at the moment of announcing a game.

Prompting health-related content as a game is followed by slightly less self-efficacy (H1), mediated by an increase of the expected difficulty (H3) between “neutral” and “somewhat difficult,” when compared with the assignment as a task. This could be interpreted as the view that games are expected to be more challenging in a negative way. Those who wish to use gaming or gamification for diabetes type II–related interventions, or more broadly in a health-related setting, should be aware of this.

There is no difference in affective state found following a game or a task prompt (H2) and no difference is found between the game and task groups in flourishing and self-esteem (H4). Together, this provides no support for the notion that a game prompt might be seen as cognitive reappraisal.

## Abbreviations

CSES: Coping Self-Efficacy Scale
PANAS: Positive and Negative Affect Schedule
PFC: Problem-Focused Coping
SISE: Single-Item Self-Esteem
SSESS: State Self-Efficacy Subscale
SUET: Stop Unpleasant Emotions and Thoughts
