# Correction: The Effect of a Health Game Prompt on Self-efficacy: Online Between-Subjects Experimental Survey

**DOI:** 10.2196/28894

**Published:** 2021-03-22

**Authors:** Priscilla Haring

In “The Effect of a Health Game Prompt on Self-efficacy: Online Between-Subjects Experimental Survey” (JMIR Serious Games 2021;9(1):e20209) the author noted one error.

In the originally published paper, the Corresponding Author address for author Priscilla Haring inadvertently listed the incorrect country as well as the incorrect country code in the author’s phone number. The originally published Corresponding Author address was listed as follows:

Priscilla Haring, MSc

Witte de withstraat 20hs

Amsterdam, 1057XW

India

Phone: 91 624813683

Email: priscillaharing@hotmail.com

This has been corrected to:

Priscilla Haring, MSc

Witte de withstraat 20hs

Amsterdam, 1057XW

Netherlands

Phone: 31 624813683

Email: priscillaharing@hotmail.com

The correction will appear in the online version of the paper on the JMIR Publications website on March 22, 2021, together with the publication of this correction notice. Because this was made after submission to PubMed, PubMed Central, and other full-text repositories, the corrected article has also been resubmitted to those repositories.

